# Inhibition of histone acetyltransferase (HAT) activity by HBZ extends beyond the p300/CBP HAT family

**DOI:** 10.1186/1742-4690-11-S1-P109

**Published:** 2014-01-07

**Authors:** Diana G Wright, Nicholas Polakowski, Isabelle Lemasson

**Affiliations:** 1 Department of Microbiology and Immunology East Carolina University, Brody School of Medicine, Greenville, NC, USA

## 

We previously reported that HTLV-1 basic leucine zipper factor (HBZ) interacts with the cellular coactivator p300 in cells derived from ATL patients. We further determined that HBZ directly binds to the histone acetyltransferase (HAT) domain of both p300 and its homologue CBP. HAT activity transfers an acetyl group to lysine residues on histone tails and transcription factors to generally upregulate transcription. We observed that the HBZ interaction with the HAT domain of p300/CBP inhibits acetylation of histones and of the tumor suppressor p53. In this study, we wanted to determine whether inhibition of HAT activity was limited to p300/CBP or extended to other HAT families. We focused on the GCN5/ p/CAF and MYST HAT families. We found that HBZ co-immunoprecipitates with both p/CAF and HBO1. These data support a recent finding that HBZ interacts with HBO1 in a yeast two-hybrid assay. Through in vitro HAT assays using recombinant proteins we found that HBZ inhibits acetylation of histone H3 and histone H4 by p/CAF and HBO1, respectively. Furthermore, HBZ reduces acetylation of p53 by p/CAF. Since both p300 and p/CAF acetylate p53 to increase its DNA-binding activity, we performed quantitative RT-PCR to evaluate expression of the p53 target genes, GADD45A and NOXA. We observed reduced mRNA levels of these genes when cells expressed HBZ. Overall these results suggest that HBZ inhibits the HAT activity of coactivators from different HAT families to contribute to transcriptional deregulation.

